# Current status of pediatric transplantation in Japan

**DOI:** 10.1186/s40560-017-0241-0

**Published:** 2017-07-20

**Authors:** Nao Nishimura, Mureo Kasahara, Kenji Ishikura, Satoshi Nakagawa

**Affiliations:** 1 Division of Critical Care Medicine, Department of Critical Care and Anesthesia, National Center for Child Health and Development, Ohkura 2-1-1, Setagaya, Tokyo, Japan; 2 Organ Transplantation Center, National Center for Child Health and Development, Ohkura 2-1-1, Setagaya, Tokyo, Japan; 3 Division of Nephrology and Rheumatology, National Center for Child Health and Development, Ohkura 2-1-1, Setagaya, Tokyo, Japan

**Keywords:** Children, Organ transplantation, Brain-dead organ donation, Living-donor organ transplantation

## Abstract

Brain-dead donor organ transplantation has been available to children in Japan since the 2010 revision of the Organ Transplant Law. Of the 50–60 brain-dead donor organ transplants performed annually in Japan, however, very few (0–4 per year) are performed in children. Again, while those receiving liver, heart, and kidney transplants are reported to fare better than their counterparts in the rest of the world, organ shortage is becoming a matter of great concern. Very few organs become available from brain-dead donors or are transplanted to adults if made available at all, with some children dying while on the brain-dead organ waiting list. Against this background, living-donor transplants, split-liver transplants, domino transplants, and hepatocyte transplants represent alternative modalities, each of which is shown to be associated with favorable outcomes. Challenges exist, include streamlining the existing framework for promoting organ donation for children and between children.

## Background

With the revision in 2010 of the Organ Transplant Law in Japan, the criteria for determining brain death in children was established, and organ donation became legal even from those less than 15 years of age. This has opened up a new venue of transplantation for children with organ failure, alongside overseas heart/other vital organ transplantation and living-donor liver/kidney transplantation, which have remained mainstays over the years.

In the meantime, a ventricular assist device (VAD) has also been approved for use as a bridge to transplantation in children with severe heart failure thought unlikely to improve with conventional pharmacotherapy, surgery, or assisted circulation.

Against this background, this review discusses the current state of organ transplantation in children as well as its future prospects.

## Main text

In Japan, the Organ Transplant Law became effective in 1997, allowing organs to be harvested from brain-dead donors. This required, however, that they have expressed their wishes for organ donation in writing while still alive and that their families agree to honor their wishes. Wishes for organ donation were only deemed valid if expressed by those 15 years of age or older, and thus, organ donation from those less than 15 years of age was not allowed. Therefore, children with end-stage organ failure had no recourse but extremely expensive overseas organ transplants or if at all available, live-donor organ transplants. Only with the revision in 2010 of the Organ Transplant Law did it become possible to determine brain death and harvest organs from brain-dead individuals 15 years of age or older despite their unclear (or unexpressed) wishes or from children less than 15 years of age if familial consent was obtained. The revision also involved prioritizing an individual’s donation preferences; individuals were now able to express wishes, while alive, regarding their possible wish to give their relatives preferential treatment in terms of who receives the organs.

Thus, of note, brain death has made heart and liver transplants possible, which cardiac arrest did not. All those who wish to receive organs from brain-dead donors are now registered with the Japan Organ Transplant Network (JOT), with the number of individuals on the waiting list shown by organ (i.e., heart, lung, liver, kidney, pancreas, and small intestine) on the JOT website. Of those less than 15 years of age on the waiting list, 31, 6, 11, 55, and 0 were registered for heart, lung, liver, kidney, and pancreas, respectively, as of October 2016. The background characteristics of these registrants are also shown by organ, which include blood type, age group, underlying disease requiring transplantation, urgency of treatment, and time on the waiting list [1].

**Fig. 1:**
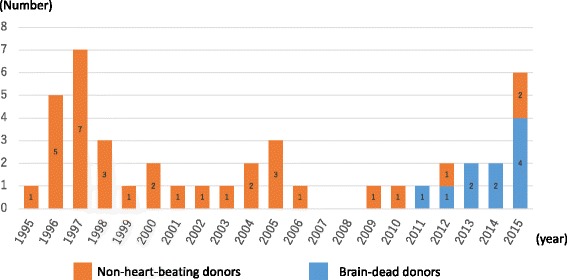
Number of pediatric deceased organ donations in Japan by year (<15 years; *n* = 43)

**Fig. 2:**
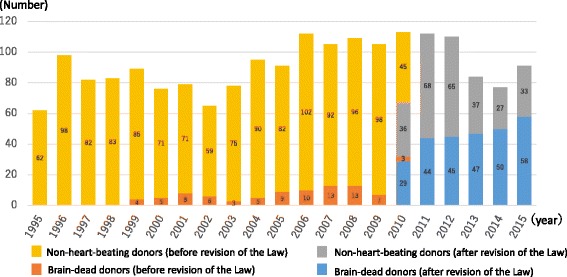
Number of deceased organ donations in Japan by year

In contrast, organs were donated from a total of 12 brain-dead individuals less than 15 years of age (including six children less than 6 years of age) between July 2010 and October 2016 or 0–4 brain-dead individuals per year (Fig. 1) [2]. This is not only true of the pediatric donors but of the entire donor population, where the number of brain-dead donors has gradually increased since the 2010 revision of the Organ Transplant Law but has leveled off at 50–60 per year. Again, the number of organs harvested from both brain-dead and cardiac arrest (non-heart-beating) donors totaled 100–110 per year between 2006 and 2012 but less than 100 in 2013 and afterwards. Thus, it is clear that brain-dead organ donors have tended to increase, but the entire organ donor population has tended to decrease in numbers over time (Fig. 2) [3].

Among the pediatric donors less than 15 years of age (*n* = 43; brain-dead, 10; non-heart-beating, 33), the causes of their death included cerebrovascular disease (*n* = 5), brain tumors (*n* = 7), respiratory diseases (*n* = 2), cardiovascular disease (*n* = 1), other internal causes (*n* = 3), head injury (*n* = 12), and other external causes (*n* = 13), and their mean age was 7.7 ± 4.5 years old (0–4 years, 12; 5–9 years, 12; and 10–14 years, 19) [2].

An examination of organ transplants performed abroad shows that transplants from deceased donors account for the largest proportion of all transplants performed in the USA, with those from brain-dead donors totaling 8000–9000 per year and those from brain-dead and non-heart-beating donors combined totaling more than 9000 per year in 2015 and 2016 [4]. Of these, over 800 per year were less than 18 years old. It is worth noting a preliminary report by an international registry which suggests that transplants from deceased donors account for as few as 0.7 per million population (PMP) in Japan in 2015, in stark contrast to 39.7 PMP in Spain, 28.5 PMP in the USA, 10 PMP in Korea, and 2 PMP in China [5].

Indeed, even after the 2010 amendment of the Organ Transplant Law, organs from deceased donors have generally remained too few in number to fill the needs of children requiring organ transplants in Japan. Thus, transplants from deceased donors remain an inadequate life-saving means for children, and as a consequence, some die while on the transplant waiting list.

To maximize the use of limited organs being made available from donors, a medical consultant system was launched in 2002 in Japan. This system involves dispatching physicians to prospective donors to ensure their hemodynamic stability, thereby improving their cardiopulmonary function. With this system in place, the number of organs transplanted per donor (OTPD) improved to as many as 6.8 in 2008, compared to 3.04 in the USA [6–8].

In what follows, we propose to describe the characteristics of representative organ transplants being performed in Japan.

## Liver transplants

### The patient numbers and the diseases to need transplantation

**Fig. 3:**
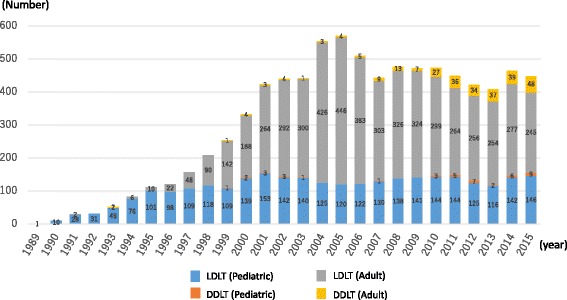
Number of liver transplants performed in Japan (*n* = 6097). *LDLT* living-donor liver transplantation, *DDLT* deceased-donor liver transplantation

According to the annual report of the Japanese Liver Transplantation Society [9], as of the end of 2015, the number of livers transplanted since the first liver transplanted at Shimane University in 1989 totaled 8387, of which 8066 and 321 were from living donors and brain-dead donors, respectively. Of the 8387 organs transplanted, 2942 were from children less than 18 years old, who accounted for a high proportion of organs harvested from all age groups. Additionally, of the 2942 organs from these children, 2897 and 45 organs were from living donors and brain-dead donors, respectively. Four to five hundred liver transplants were performed, with about 140 of these performed annually in children in 2014 and 2015 (Fig. 3) [9].

Indications for liver transplantation in children vary from those in adults and include cholestatic liver disease (mainly biliary atresia), which accounts for approximately 70% of all liver transplants performed, followed by metabolic liver disease and acute liver failure, each of which accounts for about 10% of all liver transplants performed (Table 1) [10].

**Table 1:** Indications for pediatric living-donor liver transplantation in Japan (*n* = 2224)

		Number	Percent
Cholestatic liver disease		1649	74.1
	Biliary atresia	1471	66.1
	Alagille syndrome	70	3.1
	Bayler disease	33	1.5
	Others	75	3.4
Metabolic liver disease		194	8.7
	Wilson’s disease	59	2.6
	Ornithine transcarbamylase deficiency	40	1.8
	Carbamoyl phosphate synthetase 1 deficiency	9	0.4
	Others	86	3.9
Acute liver failure		192	8.6
	Hepatitis B	9	0.4
	Drug induced	2	0.1
	Auto immune hepatitis	2	0.1
	Unknown	163	7.3
	Others	16	0.7
Neoplastic disease		66	3.0
	Hepatoblastoma	52	2.3
	Hepatocellular carcinoma	6	0.3
	Others	8	0.4
Vascular disease		32	1.4
	Congenital absence of portal vein	21	0.9
	Budd-Chiari syndrome	7	0.3
	Others	4	0.2
Re-transplantation		76	3.4
	2nd transplantation	74	3.3
	3rd transplantation	2	0.1
Others		15	0.7
Total		2224	100

### The prognosis

Japanese children less than 18 years of age receiving liver transplants are shown to fare better than adults with the survival rate being 89.4% at 1 year, 86.8% at 5 years, 84.4% at 10 years, and 80.9% at 20 years (versus 81.6% at 1 year, 72.7% at 5 years, 65.6% at 10 years, and 51.5% at 20 years) [9]; they are also shown to fare better than their counterparts in Western countries [11, 12].

### Single-center experience

**Fig. 4:**
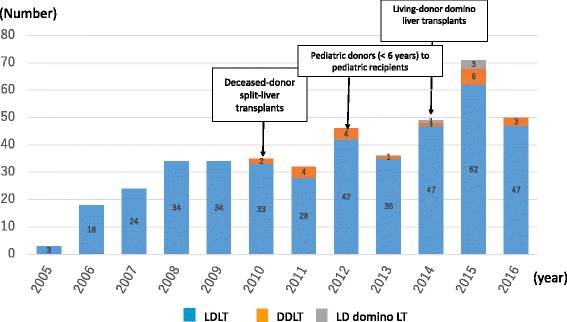
Number of liver transplants in NCCHD

As of December 2016, a total of 60–70 liver transplants were performed at our center; to date, 22 livers were transplanted from brain-dead donors (Fig. 4).

Acute liver failure is defined as “hepatic dysfunction in patients with normal livers or normal hepatic reserve, which is accompanied by a prothrombin time of less than 40% or an INR of 1.5 or higher within 8 weeks of onset of initial symptoms due to severe hepatic dysfunction” and is handled as consistent with fulminant hepatic failure presenting as acute hepatic coma of grade II or higher. While aggressive blood purification therapy is implemented in those diagnosed with fulminant hepatic failure, liver transplantation is required in those who respond poorly to such medical therapy. Indeed, fulminant hepatic failure represents a more challenging emergency than other diseases and may well represent an indication for liver transplantation from brain-dead donors. Eligibility criteria for prospective liver recipients from brain-dead donors are defined in terms of scoring based on their prognosis, underlying disease and clinical urgency (Table 2), where highly urgent and serious fulminant hepatic failure is scored 10 out of 10.

**Table 2:** Recipient selection criteria

Prognosis	Diseases	Urgency of transplantation
≤1 month	Fulminant hepatitis Citrullinemia Graft failure	10
1–3 months	Child ≥l3 points; MELD ≥25 points	8
3–6 months	Uncompensated liver cirrhosis (child C)	6
6 months–1 year	Compensated liver cirrhosis (child B)	3
≥1 year	Compensated liver cirrhosis (child A) Familial amyloid polyneuropathy	1

We previously reported our single-center experience with patients with acute liver failure [13, 14]. Of the 65 patients treated at our center for acute liver failure between November 2005 and December 2015, 54 patients (83.1%) received liver transplants.

The post-transplant rescue rate in children with fulminant hepatic failure is reported to range between 67.5 and 80% [15, 16]. Furthermore, a small number of studies compared outcomes between those less than 1 year old and those 1 year old or older, reporting that small infants fared worse than children [17–19].

Post-transplant outcomes are summarized below for the 47 children registered for liver transplants from brain-dead donors at our center between July 2010 and the end of 2012 (Table 3) [20]. A total of 25 candidates called for urgent care for their conditions (acute liver failure in nearly all cases) and had therefore been assigned the highest scores, 10 out of 10. Of these 25 patients, 7 received transplants from deceased donors and 13 from living donors, and 1 patient died while on the waiting list. A total of 10 patients, including those assigned up to 6 out of 10, received transplants from brain-dead donors. These results suggest that while pediatric transplant candidates may stand a chance of receiving transplants from brain-dead donors, medically, those candidates assigned the highest scores, 10 out of 10, based on the urgency and severity of their conditions, should not be allowed to be on the waiting list for transplants from brain-dead donors but should be considered immediate candidates for transplants from living donors.

**Table 3:** Clinical outcomes among patients on the waiting list for DDLT

Primary points	10 points (*n* = 25)	6 points (*n* = 13)	3 points (*n* = 9)
Age at enrollment in the waiting list, median (range)	10 months (11 days–17 years)	3.0 years (5 months–16 years)	4.1 years (4 months–14 years)
Underlying disease			
Acute liver failure	20		
Cholestatic liver diseases		6	5
Congenital hepatic fibrosis		4	2
Graft failure after LDLT	4		
Autoimmune liver diseases		1	2
Metabolic liver disease	1	2	
Outcomes			
Deceased-donor liver transplantation	7	3	
Living-donor liver transplantation	13	9	6

### Bridging measures and further problems

Against this background, split-liver transplants, domino transplants, and hepatocyte transplants are currently being pursued as viable options to make the most of brain-dead donor livers that remain very few in number.

Split-liver transplantation involves splitting a liver from an adult brain-dead donor into two fragments, with the larger right lobe being given to an adult recipient and the smaller left lobe (left lateral segment) being given to a pediatric recipient to accommodate his/her physique. Typically involving an adult transplant center, this approach allows a liver from an adult brain-dead donor to be transplanted in two recipients [21]. As of the end of 2014, split-liver transplants performed in Japan involved 36 of the 257 brain-dead donors who had become available and showed comparable success rates to whole-liver transplants. However, they accounted for only 6.5% (10.7% after the amendment of the Organ Transplant Law) of all transplants performed in Japan, a markedly smaller proportion compared to that in Western countries (Europe, 59.5%; USA, 16.1%), suggesting that every effort should be made to define indications for split-liver transplants involving brain-dead donors despite their limited availability.

Domino transplants initially involved patients with familial amyloid polyneuropathy as secondary donors but have recently come to involve children with maple syrup urine disease (MSUD), an inborn error of metabolism (IEM), as primary recipients and as secondary donors who, in turn, allow their livers to be removed and transplanted in non-MSUD patients [22]. While MSUD patients are associated with a deficiency in branched-chain alpha keto acid dehydrogenase resulting in impaired catabolism of the branched-chain amino acids (BCCAs), their livers may be made available for use in non-MSUD patients, in that BCCAs become catabolized by other organs than the liver in these patients. Several centers, including ours, showed that five pediatric patients received liver transplants from four MSUD patients [23].

Hepatocyte transplantation is a therapeutic modality that involves engrafting exogenous normal hepatocytes inside a partially dysfunctional host liver thereby complementing its declining function. In this modality, a proportion of the hepatocytes infused through an indwelling portal-vein catheter become engrafted and integrated and are thus thought likely to replace a deficient enzyme or complement the declining function of the host liver in acute hepatic failure. Being less invasive than liver transplantation and feasible even in neonates in whom liver transplantation proves less feasible, hepatocyte transplantation is currently being performed as bridging to transplantation in some IEMs at our center, where, to date, two neonates have undergone hepatocyte transplantation, followed by liver transplantation [24].

Given that hepatocyte transplantation alone is reported overseas to improve liver function in acute hepatic failure without recourse to liver transplantation, hepatocyte transplantation appears to hold promise as a novel option alongside the liver transplantation modalities currently available [25–27]. In contrast, living-donor transplants have remained the mainstay in Japan; while they are associated with favorable recipient results, post-transplant complications and procedure-related mortality are also reported in 8.9 and 0.03%, respectively, of the living donors [6, 28].

We believe that Japan as a whole needs to work together to further enhance the organ transplantation program for children.

Specifically, attention needs to be focused not only on minimizing complications in living donors but also on increasing the number of potential brain-dead donors, maximizing the use of organs available from brain-dead donors, developing and advancing alternative approaches to liver transplantation, and ensuring effective bridging to liver transplantation [8].

## Heart transplantation

### The patient numbers and the diseases to need transplantation

As of November 2016, the number of patients in need of heart transplants and registered with JOT for heart transplantation stands at 549, with those less than 15 years of age totaling 30 (as of January 5, 2017; see the JOT website).

According to a nationwide survey conducted by the Japanese Society of Pediatric Cardiology and Cardiac Surgery (JSPCCS) Committee for Heart Transplantation, children requiring heart transplants total some 50 annually in Japan [29]. Following approval of pediatric indications for heart transplantation, the survival rate among the pediatric recipients is shown to be 32.5% at 1 year post-transplant with the mean survival time (time to death) being 7.5 months post-transplant [30]. The time on the waiting list was approximately 900 days (more than 1000 days for 2015) even among those with severe heart disease thus representing high-priority “status 1” candidates. Statistics suggest that Japanese candidates for heart transplants are on the waiting list relatively longer than their counterparts in the rest of the world, with 31% of these patients (including adults)dying while on the waiting list [31]. Indeed, in 2015, the duration of patients who required VAD was on average, approximately 50 days in the USA, while for the 22 cases out of 44 cases who received transplantation in 2015 (50% of the cases), the duration for requiring VAD was more than 3 years in Japan [32]. Thus, patients who require transplantation in Japan need to wait much longer than their counterparts in the USA.

In Japan, VAD is intended as a bridge to transplantation and indicated for those with severe heart failure requiring circulatory assistance. Available data shows that those who have received heart transplants to date in Japan (*n* = 265) all represented the “status 1” category except 1 patient aged less than 6 years old; and, of these, 246 (92.8%) had been on VAD pre-transplant [33, 34]. In contrast, US candidates in the “status 1” category accounted for 62% of the 2200 heart transplants performed annually in the USA, with 45% of these patients shown to have been on VAD [34].

**Fig. 5:**
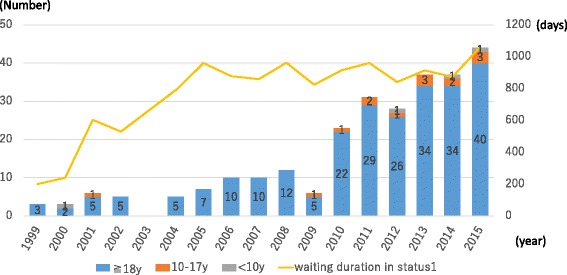
Age distribution of heart transplant recipients and mean waiting durations in Japan

**Fig. 6:**
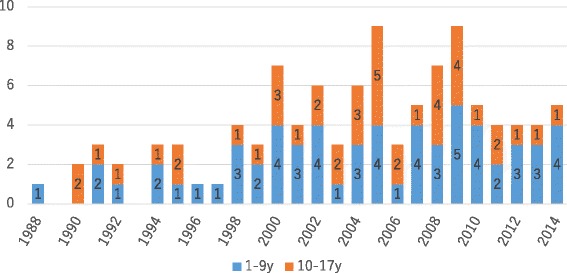
Pediatric candidates traveling overseas for heart transplants (*n* = 104)

Globally, more than 550 heart transplants are being performed annually in those less than 18 years old [35], a statistic in stark contrast to three to four heart transplants performed annually in pediatric candidates in Japan (Fig. 5). Indeed, more pediatric candidates are receiving heart transplants overseas, with 104 patients under 18 receiving heart transplants overseas by the end of 2014 (Fig. 6). However, heart transplants overseas remain extremely costly, with the pre- to post-transplant cost per transplant including outpatient visits expected ranging from 80 million to 200 million yen. They are not only becoming increasingly less feasible with very few countries available for such transplants following the Declaration of Istanbul on Organ Trafficking and Transplant Tourism in 2008 but they also give rise to associated ethical issues such as the risk of those dying on the waiting list overseas.

As of August 2016, only four facilities are available in Japan for pediatric heart transplantation in children 10 years old or younger. The indications for heart transplantation in children do not vary from those in adults, and these currently include dilated cardiomyopathy (DCM), dilated phase of hypertrophic cardiomyopathy (D-HCM), and myocardial ischemia, for which conventional treatments are unlikely to be life-saving or to prolong life expectancy, as well as any other heart disease indicated for heart transplantation by the JSPCCS Conferences on Heart Transplant Indications [34]. Besides these, potential indications include congenital heart disease unlikely to be amenable to correction with surgical interventions, which accounts for the highest proportion (54%) of all indications for heart transplantation among infants less than 1 year old overseas [35].

A total of 18 children received heart transplants in Japan as of December 31, 2015 (*n* = 18), and their underlying diseases included DCM (*n* = 14), restrictive cardiomyopathy (RCM) (*n* = 1), D-HCM (*n* = 1), post-myocarditis (*n* = 1), and DCM/RCM (*n* = 1) but no congenital heart disease. The transplants involved nine adult and nine pediatric donors, and the mean age of the recipients was 11.9 ± 5.4 years of age at the time of transplantation [33].

### The prognosis

The post-transplant 10-year survival rate among those receiving heart transplants in Japan (*n* = 222) is shown to be favorable at 89.3% compared to 53% reported for the International Society for Heart and Lung Transplantation (ISHLT) Registry, and the 10-year survival rate by age group is shown to be 100%, with one recipient dying 11 years after transplantation. Thus, Japanese heart recipients appear to fare better than those in the ISHLT Registry, where the 10-year survival rate is shown to be about 60% among all pediatric recipients, while the survival rate varies by age at transplant among those aged less than 18 years of age [36, 37].

Despite these favorable outcomes, pediatric heart transplantation as it stands in Japan has major challenges, in that the number of donors is out of proportion to that of prospective recipients. These transplant candidates are expected to be on the waiting list for as long as 2–3 years while remaining on VAD, resulting in many candidates opting to go abroad to receive transplants or face the risk of dying from complications while on the waiting list.

## Kidney transplantation

Kidney transplantation typically becomes feasible when it involves kidneys from brain-dead or non-heart-beating donors who expressed wishes to donate their organs in writing while still alive or whose wishes are unclear but their families have given consent to donate their organs (hereafter deceased donors). It differs from other forms of transplantation in that a range of treatment options are available to those with end-stage renal failure, which include ① peritoneal dialysis, ② hemodialysis, and ③ kidney transplantation, thus allowing time for alternative options to kidney transplantation.

### The patient numbers and the diseases to need transplantation

In 2015, a total of 1661 kidney transplants were performed, involving 1494 kidneys from living donors (89.8%), 63 kidneys from non-heart-beating donors who had expressed wishes to donate their organs while still alive or for whom their families had given consent to donate their organs, and 104 kidneys from brain-dead donors who had expressed wishes to donate their organs while still alive or for whom their families had given consent to donate their organs. Of these transplants, those transplanted in children less than 20 years old involved kidneys from 77 living donors and 15 deceased donors [34].

According to the report of Hattori et al., a total of 540 children developed end-stage renal failure in the 6 years between 2006 and 2011, accounting for an annual incidence of 3.5–4.7/PMP. Of note, preemptive kidney transplantation was performed in 22.3% of these patients without recourse to peritoneal dialysis or hemodialysis [38].

While the causes of end-stage renal failure in children vary by age bracket in Japan, congenital anomalies of the kidney and urinary tract are reported to be the most frequent, followed by hereditary nephropathy and focal segmental glomerulosclerosis [38, 39].

**Fig. 7:**
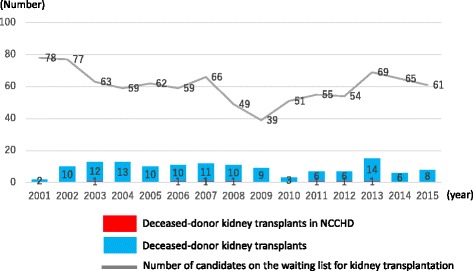
Number of pediatric deceased-donor kidney transplants performed in Japan

In recent years, a total of 90 or so transplants have been performed in children annually (involving living donors in more than 90% of cases), as well as six to eight transplants (except 2013) from deceased donors following the amendment of the Organ Transplant Law in 2010 which now allowed transplants from such donors. Very few transplants have been performed in those aged less than 16 years old and those aged 16–20 years old, despite their being assigned higher scores and thus prioritized for kidney transplantation (Fig. 7) [2].

### The prognosis

The Japan Society for Transplantation has reported on post-transplant survival rates among the transplant recipients including adults, stratifying by organ donor, living or deceased, as well as by age bracket, demonstrating improved transplant outcomes over the years, irrespective of the organ donors involved (Table 4) [34].

**Table 4:** Recipient survival by kidney transplant period in Japan

	Number	1 year (%)	5 years (%)	10 years (%)	15 years (%)
Living-donor kidney transplants					
1983–2000	7365	97.0	93.4	88.6	84.1
2001–2009	6820	98.3	96	92.7	–
2010–2014	5156	99.1	97.2	–	–
Deceased-donor kidney transplants					
1983–2000	2796	92.4	85.6	78.5	70.6
2001–2009	1323	95.9	89.2	80.8	–
2010–2014	673	97.8	93.4	–	–

Similarly, post-transplant outcomes are shown to be improving over the years, irrespective of the organ donors, according to the report of the Japan Pediatric Kidney Transplantation Clinical Statistics Subcommittee, with the 5- and 10-year engraftment rates being 96.4 and 92.3% in kidneys from living donors and 83.5 and 68% from those from deceased donors, respectively [40].

These data compare favorably with those reported overseas [41, 42].

Currently, ABO-incompatible kidney transplantation is being performed in about 10% of all kidney recipients, accounting for a greater proportion than that reported for the US counterparts (0.2%), while the modality appears to be associated with better outcomes, leading to re-transplantation being required only in 3–4% of ABO-incompatible kidney recipients, compared to 8.3% in the USA [43].

## Preferential organ allocation to pediatric transplant candidates

While the reported engraftment rates argue for the use of pediatric hearts in pediatric transplant candidates, there are reported cases of bilateral kidneys being transplanted from a pediatric donor to an adult recipient not only because of similar engraftment and survival rates reported between children and adults but because of its role in maintaining adequate renal function. Of note, however, there is an increasing focus on the preferential use of kidneys from pediatric donors in pediatric transplant candidates, prompted by the observation that the longer the transplant candidate waits, the greater the adverse influence on his/her growth and that the use of organs from pediatric donors (less than 20 years old) involves paying due regard for their parents and is deemed appropriate in pediatric transplant candidates. In liver transplantation as well, consideration is currently being given to ensuring the preferential use of livers from pediatric donors less than 18 years old to pediatric transplant candidates less than 18 years old.

## Conclusions

The present review has described the current state of organ transplantation in children. The absolute number of potential deceased, including brain-dead, donors remains small in Japan. While transplant outcomes are shown to be favorable across organs in Japan, the lack of availability of potential donors makes transplantation medicine impossible.

There are various factors leading to the limited number of brain-dead donors, not the least of which is that individuals need to recognize and address the differences between Japan’s present transplantation situation and that of foreign countries. We need to examine our existing institutions and systems and critically assess Japan’s legal definition of brain death.

In the future, it will be a challenging for the country to develop further laws and systems, but it is a vital step towards improving patient outcomes.

Despite the various factors limiting the availability of potential brain-dead donors, however, attention needs to be given to streamline the infrastructure for promoting organ transplantation in children and priority needs to be given to preferentially allocate organs to children thereby maximizing their use and saving as many children’s lives as possible. Last but not least, all practitioners need to explore in daily clinical practice how best to address the “donor shortage” issue, which is likely to remain unresolved for the foreseeable future.

## Abbreviations

BCCAs: Branched-chain amino acids
DCM: Dilated cardiomyopathy
DDLT: Deceased-donor liver transplantation
D-HCM: Dilated phase of hypertrophic cardiomyopathy
ISHLT: International Society for Heart and Lung Transplantation
JOT: Japan Organ Transplant Network
JSPCCS: Japanese Society of Pediatric Cardiology and Cardiac Surgery
LDLT: Living-donor liver transplantation
MSUD: Maple syrup urine disease
NCCHD: National Center for Child Health and Development
PMP: Per million population
RCM: Restrictive cardiomyopathy
VAD: Ventricular assist device
